# Associations between maternal risk factors of adverse pregnancy and birth outcomes and the offspring epigenetic clock of gestational age at birth

**DOI:** 10.1186/s13148-017-0349-z

**Published:** 2017-05-08

**Authors:** Polina Girchenko, Jari Lahti, Darina Czamara, Anna K. Knight, Meaghan J. Jones, Anna Suarez, Esa Hämäläinen, Eero Kajantie, Hannele Laivuori, Pia M. Villa, Rebecca M. Reynolds, Michael S. Kobor, Alicia K. Smith, Elisabeth B. Binder, Katri Räikkönen

**Affiliations:** 1 Institute of Behavioral Sciences, University of Helsinki, Helsinki, 00014 Finland; 2 Helsinki Collegium of Advanced Studies, University of Helsinki, Helsinki, 00014 Finland; 3 Department of Translational Research in Psychiatry, Department of Psychiatry and Behavioral Sciences, Max-Planck Institute of Psychiatry, Munich, 80804 Germany; 4 Department of Psychiatry and Behavioral Sciences, School of Medicine, Emory University, Atlanta, 30322 GA USA; 5 HUSLAB and Department of Clinical Chemistry, Helsinki University Hospital, Helsinki, 00029 Finland; 6 National Institute for Health and Welfare, Helsinki and Oulu, Helsinki, 00271 Finland; 7 Children’s Hospital, Helsinki University Central Hospital and University of Helsinki, Helsinki, 00029 Finland; 8 Obstetrics and Gynaecology, University of Helsinki and Helsinki University Hospital Helsinki, Helsinki, 00029 Finland; 9 Medical and Clinical Genetics and Institute for Molecular Medicine Finland, University of Helsinki and Helsinki University Hospital, Helsinki, 00014 Finland; 10 BHF Centre for Cardiovascular Science, Queen’s Medical Research Institute, University of Edinburgh, Edinburgh, EH16 4TJ UK; 11 Genetics and Molecular Biology Program, Emory University, Atlanta, 30322 GA USA; 12 Department of Gynecology and Obstetrics, School of Medicine, Emory University, Atlanta, 30322 GA USA; 13 Department of Psychiatry and Behavioral Sciences, School of Medicine, Emory University, Atlanta, GA USA; 14 Centre for Molecular Medicine and Therapeutics, BC Children’s Hospital and University of British Columbia, Vancouver, V6T 1Z4 Canada

**Keywords:** Aging, Cord blood methylation, Epigenetic clock, Gestational age, Prenatal programming

## Abstract

**Background:**

A recent study has shown that it is possible to accurately estimate gestational age (GA) at birth from the DNA methylation (DNAm) of fetal umbilical cord blood/newborn blood spots. This DNAm GA predictor may provide additional information relevant to developmental stage. In 814 mother-neonate pairs, we evaluated the associations between DNAm GA and a number of maternal and offspring characteristics. These characteristics reflect prenatal environmental adversity and are expected to influence newborn developmental stage.

**Results:**

DNAm GA acceleration (GAA; i.e., older DNAm GA than chronological GA) of the offspring at birth was associated with maternal age of over 40 years at delivery, pre-eclampsia and fetal demise in a previous pregnancy, maternal pre-eclampsia and treatment with antenatal betamethasone in the index pregnancy, lower neonatal birth size, lower 1-min Apgar score, and female sex. DNAm GA deceleration (GAD; i.e., younger DNAm GA than chronological GA) of the offspring at birth was associated with insulin-treated gestational diabetes mellitus (GDM) in a previous pregnancy and Sjögren’s syndrome. These findings were more accentuated when the DNAm GA calculation was based on the raw difference between DNAm GA and GA than on the residual from the linear regression of DNAm GA on GA.

**Conclusions:**

Our findings show that variations in the DNAm GA of the offspring at birth are associated with a number of maternal and offspring characteristics known to reflect exposure to prenatal environmental adversity. Future studies should be aimed at determining if this biological variation is predictive of developmental adversity.

**Electronic supplementary material:**

The online version of this article (doi:10.1186/s13148-017-0349-z) contains supplementary material, which is available to authorized users.

## Background

Biomarkers of cellular aging have attracted increasing attention over the past few years. Such biomarkers hold the potential to identify individuals who are at risk of aging-related diseases so that they may be offered timely, targeted preventive interventions, hopefully decades before the onset of disease. DNA methylation (DNAm) is an epigenetic mechanism characterized by the addition of one methyl group primarily to cytosine-phosphate-guanine (CpG) sites on DNA. The epigenome is known to undergo age-related changes [1–6], and specific methylated CpG sites have been strongly correlated with chronological age in humans. Hannum et al. identified 71 CpG sites in whole blood [7], and Horvath et al. identified 353 CpG sites from multiple tissues that could predict chronological age with high accuracy (*r* > 0.91). The median absolute difference between these methylation age biomarkers and chronological age has been shown to vary from between 2.9 and 4.9 years [8]. Epigenetic age acceleration (AA; higher epigenetic than chronological age) has been associated with negative health outcomes [9–11], and a recent meta-analysis of over 13,000 individuals has confirmed that it can predict all-cause mortality [12].

Epidemiological studies have shown that exposure to adverse environmental events in the prenatal period predicts increased risk of aging-related diseases [13–18]. These studies are consistent with the Developmental Origins of Health and Diseases (DOHaD) hypothesis [19], which proposes that prenatal exposures alter developmental trajectories [19]. However, it remains unclear whether epigenetic AA could identify individuals at birth who were exposed to environmental adversity in the prenatal period. A recent study assessed epigenetic AA in peripheral blood from children and adolescents based on the Hannum and the Horvath age predictors. In this study, epigenetic AA, which was higher in boys, was also associated with higher maternal body mass index (BMI) and lower maternal selenium and cholesterol levels during pregnancy, and higher birth weight of the offspring [20]. Furthermore, epigenetic AA at birth measured in DNA isolated from fetal cord blood was higher in the offspring born to mothers who had smoked during pregnancy and who were delivered by cesarean section [20].

The Hannum and Horvath epigenetic age predictors are not, however, suitable for epigenetic age estimation using fetal cord blood. The Hannum age predictor was based on whole blood taken from 19–101-year-old individuals [7] and the Horvath age predictor was based on multiple tissues taken from 0–100-year-old individuals, which includes fetal cord blood [8]. However, it should be noted that the correlation of both predictors with gestational age (GA) is nearly zero [20]. A recent study generated a novel DNAm GA predictor designed specifically for use on fetal umbilical cord blood or newborn blood spots [21]. This predictor identified methylation of 148 CpG sites that showed a strong correlation (overall *r* = 0.91) with ultrasound-based GA [21]. The average absolute difference between the predicted DNA methylation GA and GA was 1.49 weeks [21]. Using fetal umbilical cord blood samples, another recent study identified 96 and 58 CpG sites, which however correlated less strongly with ultrasound-based (*r* = 0.81) and last menstrual period-based GA (*r* = 0.71) [22]; Only 23 of the ultrasound- and last menstrual period-based GA predictor CpG sites overlapped [22].

In the present study, we studied the DNAm GA predictor at birth based on fetal umbilical cord blood methylation data as developed by Knight et al. [21]. We tested whether DNAm GA is associated with a number of maternal and offspring characteristics known to reflect a suboptimal prenatal developmental milieu of the offspring. These characteristics include maternal pre-pregnancy risk factors of pre-eclampsia and intrauterine growth restriction, maternal pregnancy disorders, maternal treatment with antenatal corticosteroids, parity and mode of delivery, as well as newborn body size at birth, cord blood pH, and Apgar score. For comparison, we also present these associations with the Horvath epigenetic age, which shares only six overlapping CpG sites with the DNAm GA predictor of Knight et al. [21].

## Results

**Fig. 1:**
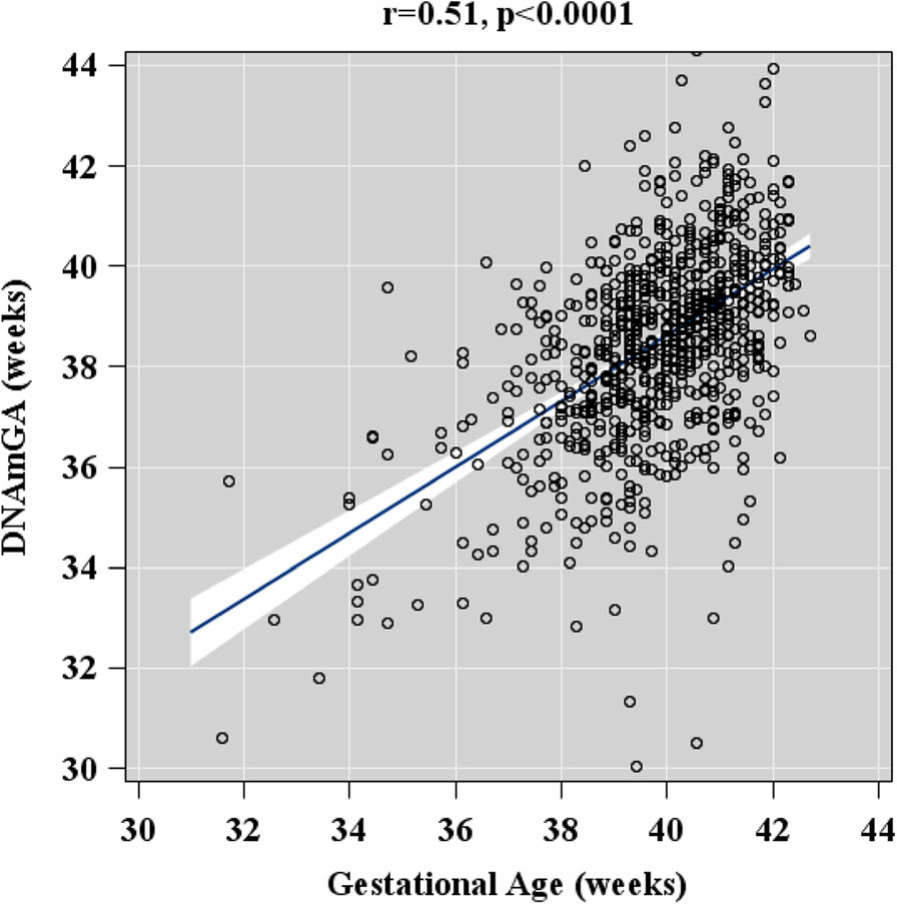
Association between offspring DNAm gestational age (GA) at birth based on cord blood methylation data and offspring chronological ultrasound-based GA at birth. *Scatterplot* shows regression line and 95% confidence intervals. *p* value refers to the significance level of the association

The associations of maternal and neonatal characteristics with the raw DNAm GA difference (arithmetic difference between DNAm GA and GA) and with the DNAm GA residual (the residual from a linear regression of DNAm GA on GA) were tested in 814 women and their singleton neonates participating in the Prediction and Prevention of Pre-eclampsia and Intrauterine Growth Restriction (PREDO) study (Additional file 1: Figure S1). The mean GA at birth of this cohort was 39.76 (standard deviation (SD) 1.60; median 39.86; range 31.0–42.71) weeks, and the mean DNAm GA at birth was 38.45 (SD 2.05; median 38.60; range 28.50–47.13) weeks. There was a positive correlation between the DNAm GA and GA (*r* = 0.51; *p* < 0.0001; Fig. 1). The average absolute difference (arithmetic difference between DNAm GA absolute values and GA) between DNAm GA and GA was 1.78 (SD 1.41; median 1.51) weeks, and the raw mean difference was −1.32 (SD 1.85; median −1.36; range −10.64–6.97) weeks. There was a weak negative correlation between the raw DNAm GA difference and GA (*r* = −0.30; *p* < 0.0001). DNAm GA residual did not correlate with GA (*r* = 0, *p* = 1.0).

Pearson correlations between the Horvath DNAm age and our values were as follows: GA 0.03 (*p* = 0.32), DNAm GA 0.11 (*p* = 0.002), raw DNAm GA difference 0.09 (*p* = 0.01), and DNAm GA residual 0.11 (*p* = 0.01).

Characteristics of the study population are presented in Table 1.

**Table 1:** Characteristics of the study population (*N* = 814)

Maternal characteristics	Mean (SD) or *N* (%)
Pre-pregnancy risk factors^a^	
Maternal age at delivery, years	33.3 (5.8)
Below 20 years, yes	22 (3.0%)
Above 40 years, yes	115 (14.1%)
Pre-eclampsia in previous pregnancy, yes	178 (21.9%)
Intrauterine growth estriction in previous pregnancy, yes	85 (10.4%)
Gestational diabetes in previous pregnancy, yes	84 (10.3%)
Diet treated	75 (9.2%)
Insulin treated	9 (1.1%)
Pre-pregnancy body mass index kg/m^2^	27.4 (6.4)
≥30 kg/m^2^	287 (35.3%)
Pre-pregnancy chronic hypertension, yes	109 (13.4%)
Pre-pregnancy type 1 diabetes, yes	12 (1.5%)
Pre-pregnancy systemic lupus erythematosus, yes	2 (0.3%)
Sjögren’s syndrome, yes	11 (1.4%)
Previous pregnancy with fetal demise (>22 gestational weeks or over 500 g), yes	28 (3.4%)
Number of known pre-pregnancy risk factors	
No known pre-pregnancy risk factors	79 (9.7%)
1 or 2 pre-pregnancy risk factors	696 (85.5%)
3 or more pre-pregnancy risk factors	31 (3.8%)
Data not available	8 (1.0%)
Pregnancy disorders	
Gestational diabetes, yes	183 (22.5%)
Diet treated	147 (18.1%)
Insulin treated	36 (4.4%)
Data not available on gestational diabetes treatment	2 (0.2%)
Data not available on gestational diabetes diagnosis	1 (0.1%)
Hypertension spectrum pregnancy disorders, yes	
Gestational hypertension	80 (9.8%)
Pre-eclampsia	61 (7.5%)
Early pre-eclampsia (diagnosis <34 weeks of gestation)	53 (6.5%)
Late pre-eclampsia (diagnosis ≥34 weeks of gestation)	8 (1.0%)
Non-severe pre-eclampsia	42 (5.2%)
Severe pre-eclampsia	19 (2.3%)
Chronic hypertension	134 (16.5%)
Data not available	1 (0.1%)
Other characteristics	
Education level	
Lower secondary or less	359 (44.1%)
Upper secondary	184 (22.6%)
Tertiary	248 (30.5%)
Data not available	23 (2.8%)
Parity	
Primiparous	247 (30.3%)
Multiparous	566 (69.5%)
Data not available	1 (0.1%)
Smoking during pregnancy	
Non-smoker	780 (95.8%)
Quit during first trimester	26 (3.2%)
Smoked throughout the pregnancy	8 (1.0%)
Data not available	0
Alcohol use during pregnancy	
No	588 (72.2%)
Yes	117 (14.4%)
Data not available	109 (13.4%)
Mode of delivery	
Vaginal	640 (78.6%)
Cesarean	173 (21.3%)
Data not available	1 (0.1%)
Antenatal betamethasone treatment	
No	750 (92.1%)
Yes (*n* = 1 for 12 mg/24 h, *n* = 22 for 24 mg/24 h, *n* = 1 for 48 mg/24 h, *n* = 11 for no information on dose)	35 (4.3%)
Timing of antenatal betamethasone treatment, number of days before birth	33.4 (27.1)
30 days or less before delivery	14 (1.7%)
30 days or more before delivery	13 (1.6%)
Data not available	29 (3.6%)
Neonatal characteristics	
Sex	
Girls	384 (47.2%)
Boys	430 (52.8%)
Data not available	0
Gestational age at birth, weeks	39.76 (1.60)
Data not available	0
DNA methylation gestational age, weeks	38.45 (2.05)
Data not available	0
Raw epigenetic gestational age difference, DNA methylation gestational age-gestational age	−1.32 (1.85)
Data not available	0
Absolute epigenetic gestational age difference, DNA methylation gestational age-gestational age in absolute values	1.78 (1.41)
Data not available	0
Horvath DNA methylation age at birth, weeks	9.77 (19.51)
Data not available	0
Birth weight, g	3549 (546)
Small for gestational age, yes^b^	23 (2.8%)
Data not available	1 (0.1%)
Birth length, cm	50 (2)
Small for gestational age, yes^b^	21 (2.6%)
Data not available	1 (0.1%)
Head circumference, cm	35 (2)
Small for gestational age, yes^b^	14 (1.7%)
Data not available	2 (0.3%)
Ponderal index, kg/m^3^	27.8 (2.7)
Data not available	1 (0.1%)
Placenta weight, g	615 (134)
Cord blood pH	
Arterial	7.26 (0.09)
Venous	7.31 (0.08)
Apgar score	
9–10	611 (75.1%)
7–8	145 (17.8%)
≤6	47 (5.8%)
Data not available	11 (1.4%)

^a^Pre-pregnancy risk factors served as inclusion criteria for the study as described [39]

^b^Small for gestational age indicates birth size for sex and gestational age SD ≤ −2 according to Finnish growth references [23]

### Maternal characteristics during pregnancy and offspring DNAm GA at birth

**Fig. 2:**
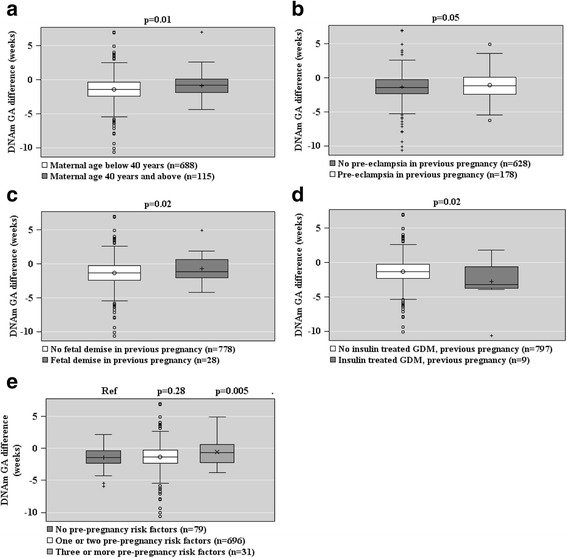
Associations between maternal pre-pregnancy risk factors of pre-eclampsia and intrauterine growth restriction (panels **a**–**e**) and raw epigenetic gestational age (GA) difference (DNAm GA-GA) of the offspring at birth based on fetal cord blood methylation data. Associations are adjusted for cell-type composition and population stratification estimated with two multi-dimensional scaling components based on genome-wide data. Data shown are median, interquartiles, and range. *p* values refer to group differences. *Ref* referent group

**Fig. 3:**
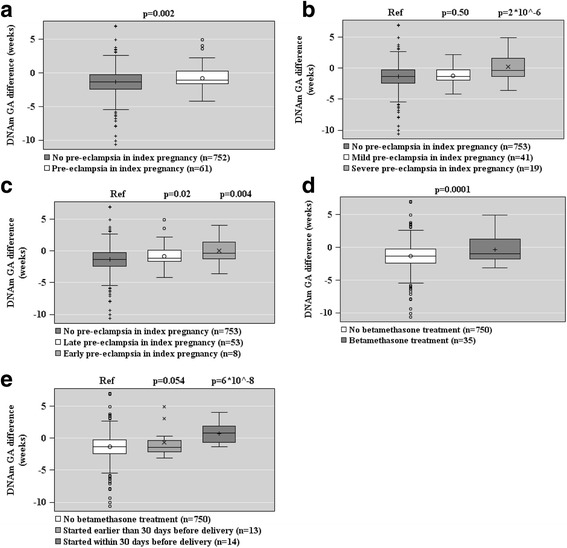
Associations between maternal pregnancy disorders in the index pregnancy and other maternal characteristics (panels **a**–**e**) and raw epigenetic gestational age (GA) difference (DNAm GA-GA) of the offspring at birth based on fetal cord blood methylation data. Associations have been adjusted for cell-type composition and population stratification estimated with two multi-dimensional scaling components based on genome-wide data. Data shown are median, interquartiles, and range. *p* values refer to group differences. *Ref* referent group

**Fig. 4:**
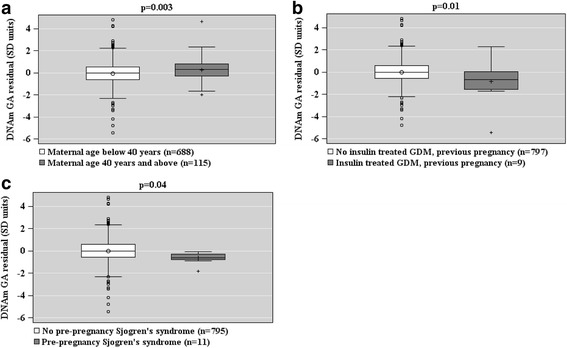
Associations between maternal pre-pregnancy risk factors of pre-eclampsia and intrauterine growth restriction (Panels **a**–**c**) and epigenetic gestational (GA) residual (the residual from a linear regression of DNAm GA on GA) of the offspring at birth based on fetal cord blood methylation data. Associations are adjusted for cell-type composition and population stratification estimated with two multi-dimensional scaling components based on genome-wide data. Data shown are median, interquartiles, and range. *p* values refer to group differences

Figures 2, 3, and 4 show the associations between maternal characteristics during pregnancy with the raw DNAm GA difference and the DNAm GA residual of the offspring. The regression models are adjusted for cellular heterogeneity and population stratification. When based on the raw DNAm GA difference, GAA was associated with a maternal age of above 40 years at delivery, pre-eclampsia in a previous pregnancy, fetal demise in a previous pregnancy, and having three or more of the pre-pregnancy risk factors for pre-eclampsia and intrauterine growth restriction (Fig. 2). GAA was also associated with the presence, onset, and severity of maternal pre-eclampsia in the index pregnancy and maternal treatment with betamethasone in the index pregnancy, particularly if the treatment was started a maximum of 30 days before delivery (Fig. 3). GAD was associated with insulin-treated GDM in a previous pregnancy (Fig. 2).

When based on the DNAm GA residual, GAA was associated with a maternal age of above 40 years at delivery, and GAD with insulin-treated GDM in a previous pregnancy and maternal Sjögren’s syndrome (Fig. 4).

Additional file 2: Table S1 shows the unstandardized regression coefficients and 95% confidence intervals for the associations depicted in Figs. 2, 3, and 4 and for the associations between the other tested maternal characteristics during pregnancy and offspring DNAm GA at birth. Additional file 2: Table S2 shows that all of the significant associations remained significant when additionally adjusted for the birth weight SD score based on Finnish national growth references [23].

Additional file 2: Table S3 shows the associations between maternal characteristics and the offspring’s Horvath epigenetic age at birth.

### Offspring characteristics and DNAm GA at birth

**Fig. 5:**
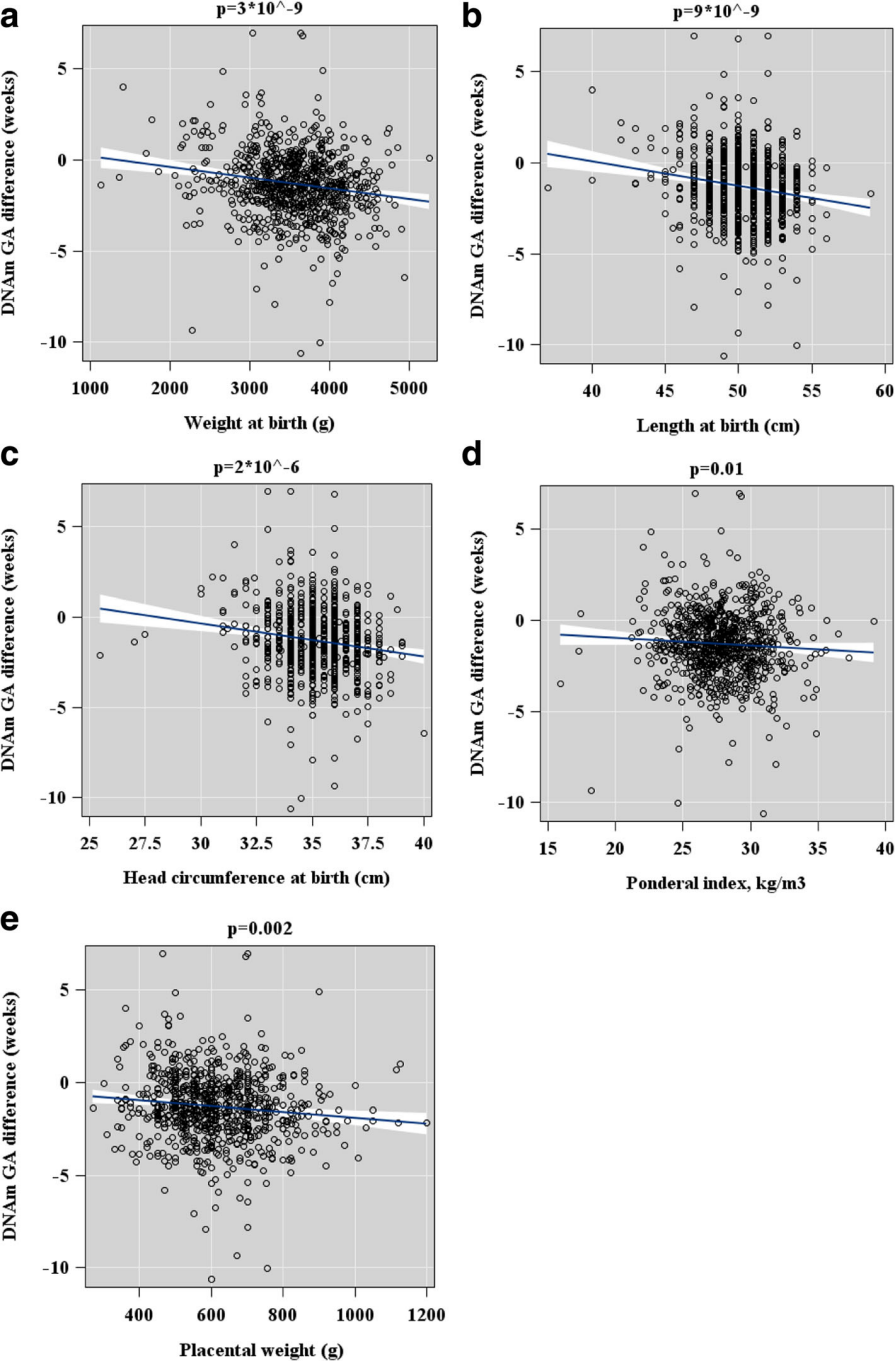
Associations between offspring anthropometry (panels **a**–**d**) and placental weight at birth (panel **e**) and raw epigenetic gestational (GA) difference (DNAm GA-GA) of the offspring at birth based on fetal cord blood methylation data. Associations have been adjusted for cell-type composition, population stratification estimated with two multi-dimensional scaling components based on genome-wide data, and neonatal sex. *Scatterplots* show regression lines and 95% confidence intervals. *p* values refer to significance levels of the associations

**Fig. 6:**
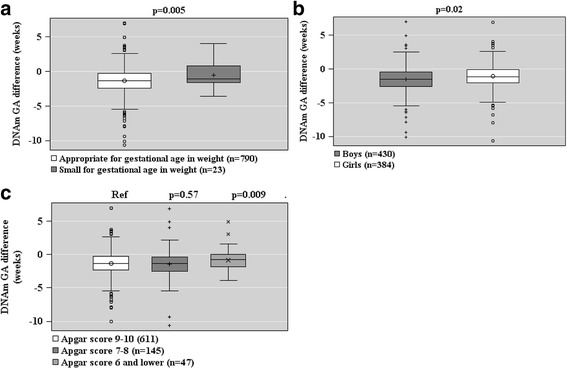
Associations between offspring small for gestational age (GA) weight at birth (panel **a**), sex (panel **b**), and Apgar score (panel **c**), and raw DNAm GA difference (DNAm GA-GA) of the offspring at birth based on fetal cord blood methylation data. Associations are adjusted for cell-type composition and population stratification estimated with two multi-dimensional scaling components based on genome-wide data. Data shown are median, interquartiles, and range. *p* values refer to group differences. *Ref* referent group

GAA, based on the raw DNAm GA difference, was associated with lower birth weight, birth length, ponderal index at birth, birth head circumference, placental weight (Fig. 5), being a lower birth weight for GA (continuous and being small-for-gestational-age, <−2 SD), a lower 1-min Apgar score, and female sex (Fig. 6). All models were adjusted for cellular heterogeneity, population stratification, and additionally for sex in the analyses of the offspring birth anthropometry.

**Fig. 7:**
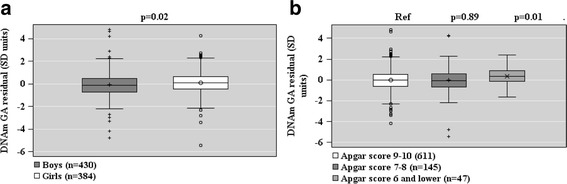
Associations between offspring sex (panel **a**) and Apgar score (panel **b**) and epigenetic gestational age (GA) residual (the residual from a linear regression of DNAm GA on GA) of the offspring at birth based on fetal cord blood methylation data. Associations are adjusted for cell-type composition and population stratification estimated with two multi-dimensional scaling components based on genome-wide data. Data shown are median, interquartiles, and range. *p* values refer to group differences. *Ref* referent group

When based on the DNAm GA residual, GAA was associated with a lower 1-min Apgar score and female sex (Fig. 7). Additional file 2: Table S4 shows the unstandardized regression coefficients and 95% Confidence Intervals for the associations depicted in Figs. 5, 6, and 7 and for the associations between the other tested offspring characteristics and offspring DNAm GA at birth.

Additional file 2: Table S5 shows the associations between offspring characteristics and the offspring Horvath epigenetic age at birth.

## Discussion

We show that a number of maternal and offspring characteristics known to reflect the offspring’s exposure to environmental adversity during the prenatal period were associated with variations in the offspring’s DNAm GA at birth. These characteristics are expected to influence the newborn developmental stage and fetal organ and tissue maturation. Characteristics associated with DNAm GAA included a number of pre-eclampsia and intrauterine growth restriction pre-pregnancy risk factors: maternal age of over 40 years, pre-eclampsia and fetal demise in a previous pregnancy, and having a higher number of pre-pregnancy risk factors of pre-eclampsia and intrauterine growth restriction that were measured in this study. DNAm GAA was also associated with maternal pre-eclampsia in the index pregnancy and treatment with antenatal betamethasone, which hastens fetal lung maturation and maturation of some other tissues [24]. It was also associated with a smaller body size at birth and being born small-for-gestational age, lower 1-min Apgar score, and female sex. Furthermore, our findings show that DNAm GAD was associated with insulin-treated GDM in a previous pregnancy and Sjögren’s syndrome. These findings were more accentuated when the DNAm GA calculation was based on the raw difference between DNAm GA and GA (which shared 9% of variance with GA) than on the residual from linear regression of DNAm GA on GA (which removed the effect of GA entirely from DNAm GA, and hence, was uncorrelated with GA). Our findings emphasize that neonates exposed to prenatal environmental adversity show differences at birth in their DNAm GA and GA, confirmed by early pregnancy ultrasound.

This conclusion is in agreement with a recent study, which first generated the used biomarker for the epigenetic clock for GA at birth and tested associations between maternal socioeconomic status and newborn birth weight with DNAm GA [21]. However, in contrast to our findings, the previous study showed that DNAm GAD was associated with mother’s socioeconomic disadvantage during pregnancy and the offspring’s lower birth weight [21]. Also in contrast to our report, they report no sex differences in the median errors between DNAm GA and GA, i.e., boys and girls did not differ in their DNAm GA at birth [21]. Another recent study, which used the DNAm age predictors of Horvath and Hannum on cord blood methylation data, also concluded that prenatal adversity is associated with epigenetic age at birth [20]. Yet, of the 20 different maternal and neonatal characteristics tested in that study, including maternal age, education, alcohol use during pregnancy, body mass index (BMI), parity, birth weight, and sex, only maternal smoking during pregnancy and cesarean section were associated with epigenetic AA, independent of GA at birth. However, the Horvath and Hannum epigenetic age did not correlate with the newborn chronological GA [20, 21]. This supports our study, as we found that the Horvath epigenetic age at birth based on cord blood methylation data was uncorrelated with the newborn GA. So, while the overall conclusion from these prior studies is similar to ours, discrepancy in the direction of associations indicates that future studies are still needed. Yet, in effect size, the prior findings and those of ours do not greatly differ. For instance, in the Knight et al. study [21], offspring birth weight accounted for 2% of the variance of the DNAm GA, when accounting for GA and the other covariates; in our study, birth weight was unrelated with DNAm GA adjusted for GA, cellular heterogeneity, and population stratification, but child’s sex accounted for 1% and Apgar score accounted for 1% of the variance of the DNAm GA. In the Simpkin et al. study [20], maternal smoking during pregnancy and cesarean section delivery explained less than 1% of the variance of offspring’s Horvath age at birth. In our study, these characteristics and the offspring’s Horvath age at birth were generally unrelated. Thus, future studies will need to determine to what extent the different associations and their direction reflect differences between the studies due to tissue type (cord blood plus newborn blood spots vs cord blood only), cellular composition of samples, fetal cord blood contamination with maternal blood, and population genetic structure. These factors were only taken into account in our study. Differences may also relate to sample characteristics: 89.3% of women in our sample had at least 1 of the 10 pre-pregnancy risk factors of pre-eclampsia and intrauterine growth restriction. This resulted in greater variation in many maternal and offspring characteristics, including GA and DNAm GA, which are slightly different in this study from the study conducted by Knight et al. [21].

Both DNAm GAA and GAD could be conceived as indicators of risk. The increased risk of future adverse outcomes associated with DNAm GAA is congruent with findings in children and adults showing that epigenetic age acceleration is associated with a number of adverse characteristics including higher BMI [25], lower physical and cognitive fitness [26], and increased mortality [9, 10, 12]. Lower developmental maturity, as indicated by DNAm GAD, is consistent with the DOHaD hypothesis and findings from previous studies showing an increased risk of aging-related diseases in individuals exposed to prenatal environmental adversity associated with pre-term birth [27–31]. Hence, the associations with DNAm GAA or GAD may serve as summary indicators of epigenetic programming and indicate increased risk for adverse outcomes later in life. As suggested by Knight et al. [21], an alternative explanation for the difference between DNAm GA and GA may be the variable nature of the clinical GA estimation. Yet, in our sample, this explanation may be less likely as GA estimation in all women was based on ultrasound performed between 12 + 0 and 13 + 6 gestational weeks + days.

However, it is important to note that when DNAm GA was based on the residual, which removed the effect of GA on DNAm GA entirely, many of the maternal and neonatal characteristics were no longer associated with DNAm GA. Only a maternal age of above 40 years, lower 1-min Apgar score, and female sex were associated with residual GAA and insulin-treated GDM in a previous pregnancy and maternal Sjögren’s syndrome were associated with residual GAD. If DNAm GA reflects developmental maturity, we cannot rule out it being independent of various environmental factors, which may alter the maturational process. Associations may become more evident later in childhood as the variation in methylation increases [3, 7, 32–34].

In our study, we used two measurements: raw DNAm GA and residual DNAm GA. Residual DNAm GA was corrected for any confounding effect of GA on DNAm GA and hence did not correlate with GA. However, if we had only focused on this variable, any finding which was associated with both GA and DNAm GA would be omitted, and hence, our analysis might have been too restrictive. Therefore, we decided to also present findings from the raw DNAm GA in our study. These two measures of DNAm GAA and GAD might serve different applications. The DNAm GA residual might be a more appropriate measure for testing hypotheses on a population level. As it is dependent on population characteristics, it may not have clinical, individual level utility unless population level “DNAm GA standards” become available, analogous to national references for fetal growth [23]. The raw DNAm GA difference may be a more useful and clinically relevant measure for individual level assessments.

### Strengths

The main strength of our study is the use of a well-characterized prospective, ethnically homogenous cohort with data on pre-pregnancy risk factors of pre-eclampsia and intrauterine growth restriction, pregnancy disorders validated by a clinical jury consisting of two qualified physicians and a nurse, and data on other maternal and neonatal characteristics extracted from both medical records and the Finnish Medical Birth Register (MBR) [35]. Our sample was enriched for women with known risk factors for pre-eclampsia and intrauterine growth restriction. This resulted in greater variation of the pre- and neonatal characteristics, thus increasing the possibility of being able to detect their effects on the DNAm GA predictor. Furthermore, clinical GA estimation was based on an ultrasound scan conducted in all women between 12 + 0 and 13 + 6 weeks + days of gestation. DNAm GA was estimated from fetal umbilical cord blood, and we applied novel methods to account for any contamination of the samples by maternal blood. A number of studies have shown that cellular heterogeneity [36], and genetic variation in the population structure [37, 38], can influence epigenetic profiles. Therefore, we removed any potential effects of cell type heterogeneity using bioinformatics methods and corrected for population structure using principal components derived from genome-wide genotypes.

### Limitations

Several strengths of this study are also limitations. The ethnic homogeneity of our sample may preclude generalizations for other ethnic groups. Our inclusion criteria, which resulted in enrichment of pregnancy disorders in the study population and increased statistical power to detect their effects, also limit generalizability of these findings to women without such risk factors. Finally, our findings are limited to one tissue type; therefore, we could not test cross-tissue correlations. It is also important to note that for some prenatal characteristics, e.g., for insulin-treated diabetes in a previous pregnancy, maternal Sjögren’s syndrome, less than ten pairs of women and neonates were present in the risk group. Therefore, although we observed significant associations, they should be interpreted with caution and need to be replicated. Finally, while maternal pre-eclampsia in the index pregnancy, maternal treatment with betamethasone, lower birth weight and length, and lower placental weight remained associated with GAA after Bonferroni correction for multiple testing, this correction may be too conservative as the tested associations were not independent.

## Conclusions

Our findings show that a number of maternal and offspring characteristics known to reflect the offspring’s prenatal environment are associated with variations in the offspring’s DNAm GA at birth based on data from cord blood DNA methylation. Whether this variation is predictive of developmental outcomes in later life is the subject of ongoing studies.

## Methods

### Study population

Data were taken from the PREDO study, which is a longitudinal multicenter pregnancy cohort study of Finnish women and their singleton children born alive between 2006 and 2010 [39]. We recruited 1079 pregnant women, of whom 969 had 1 or more, and 110 had none of the known risk factors for pre-eclampsia and intrauterine growth restriction (Table 1). The recruitment took place in arrival order when these women attended the first ultrasound screening at 12 + 0–13 + 6 weeks + days of gestation in 1 of the 10 hospital maternity clinics participating in the study. The cohort profile [39] contains details of the study design, inclusion criteria, and all the data that are available. Additional file 1: Figure S1 shows a flowchart of the 814 mother-offspring pairs with data available for the current study.

### Offspring DNA methylation, GA, and DNAm GA at birth

Fetal cord blood samples were collected according to standard procedures. DNA was extracted at the National Institute for Health and Welfare, Helsinki, Finland, and the Department of Medical and Clinical Genetics, University of Helsinki, Finland. Methylation analyses were performed at the Max Planck Institute of Psychiatry in Munich, Germany. DNA was bisulphite-converted using the EZ-96 DNA Methylation kit (Zymo Research, Irvine, CA). Genome-wide methylation status of over 485,000 CpG sites was measured using the Infinium Human Methylation 450 BeadChip (Illumina Inc., San Diego, USA) according to the manufacturer’s protocol. The arrays were scanned using the iScan System (Illumina Inc., San Diego, USA). The quality control (QC) pipeline was set up using the R-package minfi (http://bioconductor.org/packages/release/bioc/html/minfi.html). The samples were excluded if they were duplicates, outliers in the median intensities, and because of sex discrepancy. Furthermore, any probes on chromosome X or Y, cross-hybridizing probes as well as probes containing SNPs, and CpGs with a detection *P* value > 0.01 in at least 50% of the samples, or maternal blood contamination were excluded. Maternal blood contamination was tested using DNAm data at 10 CpGs independently identified as differentially methylated between cord and adult blood and indicative of maternal blood contamination (paper under review). The samples with DNAm values above the previously identified thresholds at five or more of these CpGs were considered contaminated and removed from all future analyses. The final dataset contained 428,619 CpGs. Additional file 1: Figure S1 shows that of the 876 samples available for these analyses, 51 were excluded. Methylation beta-values were normalized using the funnorm function and incorporating the first ten principal components from the internal control probes. To check for batch effects, principal components were computed on these beta values. Two batches, i.e., slide and well, were significantly associated to the main principal components and were removed iteratively using the combat package.

DNAm GA was calculated using the method published by Knight et al. [21] and was based on the methylation profile of 148 selected CpGs.

We calculated a raw DNAm GA difference by subtracting the chronological GA assessed at the first ultrasound screening conducted at 12 + 0–13 + 6 weeks + days of gestation from the predicted DNAm GA. DNAm GA residual was extracted from a linear regression of predicted DNAm GA on ultrasound-based GA.

### Offspring cord blood cell counts at birth

Seven cell types (nucleated red blood cells, granulocytes, monocytes, natural killer cells, B cells, CD4(+)T cells, and CD8(+)T cells) were estimated from cord blood methylation using the method of Bakulski et al. [40] which was also incorporated in the R-package minfi.

### Offspring genome-wide genotyping and multi-dimensional scaling analysis

Genotyping was performed on Illumina Human Omni Express Exome Arrays containing 964,193 SNPs. Only markers with a call rate of at least 98%, a minor allele frequency of 1%, and a *P* value for deviation from Hardy-Weinberg-Equilibrium (*P* > 1.0 e-06) were kept in the analysis. After QC, 587,290 SNPs were available. Any sample pair with IBD estimates >0.125 was checked for relatedness. For most pairs, high IBD estimates could be explained due to partly African origin. As we corrected for admixture in our analyses, these samples were kept except for one pair. For this pair, the high IBD estimate could not be resolved and the other one of this pair was excluded from further analysis. The samples showing discrepancies between phenotypic and genotypic sex were removed. We also checked for heterozygosity outliers, but found none. In total, we genotyped 996 samples of which 13 were excluded. Of the samples with DNA methylation data, eight were excluded based on the above criteria (Additional file 1: Figure S1).

We performed multi-dimensional scaling (MDS) analysis on the IBS matrix of quality-controlled genotypes. Apart of those samples with African admixture, no outliers were detected. The first two components depict this admixture and were included as covariates in the regression analysis.

### Maternal characteristics during pregnancy

Pre-pregnancy risk factors of pre-eclampsia and intrauterine growth restriction were derived from medical records screened for by trained study nurses or clinic personnel at maternity clinics of study hospitals at enrolment into the study. These pre-pregnancy risk factors are listed in Table 1.

Pregnancy disorders, derived from hospital records and further verified by a clinical jury [39, 41], included gestational diabetes, which was defined as fasting, 1- or 2-h plasma glucose during a 75-g oral glucose tolerance test ≥5.1, 10.0 or 8.5 mmol/L, respectively, that emerged or was first identified during pregnancy, and further categorized according to treatment as diet or insulin treated; gestational hypertension, which was defined as systolic blood pressure ≥140 mmHg and/or diastolic blood pressure ≥90 mmHg on ≥2 occasions at least 4 h apart in a woman who was normotensive before 20 weeks of gestation; pre-eclampsia, which was defined as systolic blood pressure ≥140 mmHg and/or diastolic blood pressure ≥90 mmHg on ≥2 occasions at least 4 h apart with proteinuria ≥300 mg/24 h. Pre-eclampsia diagnosis was further divided into early (diagnosis before 34 weeks of gestation) and late pre-eclampsia (diagnosis 34 weeks of gestation or later), and also into severe (blood pressure ≥160 mmHg systolic and/or ≥110 mmHg diastolic and/or proteinuria ≥5 g/24 h) and non-severe pre-eclampsia (blood pressure 140–159.9 mmHg systolic and/or 90–109.9 mmHg diastolic and/or proteinuria 0.3–4.9 g/24 h); chronic hypertension was defined as systolic/diastolic blood pressure ≥140/90 mmHg or antihypertensive medication before 20 weeks of gestation (in 24 out of 135 women chronic hypertension was diagnosed during pregnancy). In addition to pre-pregnancy obesity, data on maternal pre-pregnancy BMI calculated from measured weight and height at the first antenatal clinic visit at 8 + 4 (SD 1 + 3) weeks + days were derived from the Finnish Medical Birth Register (MBR) [35] and data on weight change during pregnancy from medical records.

Information on the antenatal corticosteroid (betamethasone) treatment was derived from hospital records (one woman received half a standard dose, i.e., 12 mg/24 h, 22 women received one standard dose of 24 mg/24 h, and one woman received two standard doses totalling 48 mg/24 h), and timing of exposure was further defined as the number of days before birth (over 30 vs 30 days or less before birth).

Maternal smoking during pregnancy (non-smoker, quit during first trimester, smoked throughout), parity (primiparous vs multiparous) and mode of delivery (vaginal delivery vs caesarian section) were derived from the MBR, and alcohol use (yes vs no) and education level (lower secondary or less, upper secondary, tertiary) were reported at 12 + 0–13 + 6 gestational weeks + days.

### Offspring characteristics at birth

Weight (kg), length (cm), head circumference (cm), fetal cord blood venous and arterial pH, and 1-min Apgar score were measured at birth, and the birth ponderal index (kg/m^3^) was calculated. We further divided birth weight and length into normal and small (≤ − 2SD) for GA using Finnish national growth references [23].

### Statistical analysis

We tested the associations between maternal and offspring characteristics with the raw DNAm GA difference, DNAm GA residual, and Horvath epigenetic age with linear regressions. All models were adjusted for cell-type composition and population stratification estimated with two multi-dimensional scaling components based on genome-wide data. Maternal characteristic data were further adjusted for birth weight SD score and neonatal anthropometric data for child’s sex. Unstandardized regression coefficients and 95% confidence intervals (CI) represent effect sizes in weeks (raw DNAm GA difference and Horvath epigenetic age) and SD units with a mean of 0 and SD 1 (DNAm GA residual). Nominal 2-tailed *p* values are given in the tables and Bonferroni-corrected *p* value threshold reaching a level of *p* < 0.05 in footnotes. All statistical analyses were performed using SAS 9.4 (SAS Institute, Inc., Cary, NC, USA).

## Additional file

Additional file 1: Figure S1. Flowchart of the study participants and sample attrition. (PPTX 38 kb)
Additional file 2: Table S1. Associations between maternal characteristics during pregnancy and offspring DNAm GA at birth based on cord blood methylation data. Table S2. Associations between maternal characteristics during pregnancy and offspring DNAm GA at birth based on cord blood methylation data when additionally adjusting for offspring birth weight SD score at birth*. Table S3. Associations between maternal characteristics and the offspring Horvath epigenetic age at birth based on cord blood methylation data*. Table S4. Associations between offspring characteristics and DNAm GA at birth based on cord blood methylation data. Table S5. Associations between offspring characteristics and the offspring Horvath epigenetic age at birth based on cord blood methylation data*. (DOCX 65 kb)

## Abbreviations

DNAm GA: DNA methylation gestational age
DOHaD: Developmental Origins of Health and Disease
GA: Gestational age
GAA: Gestational age acceletaion
GAD: Gestational age deceleration
GDM: Gestational diabetes mellitus
PREDO: Prediction and Prevention of Pre-eclampsia and Intrauterine Growth Restriction Study
SD: Standard deviation
SGA: Small for gestational age
