# Supplementary material for: Associations between maternal risk factors of adverse pregnancy and birth outcomes and the offspring epigenetic clock of gestational age at birth
Source: Clin Epigenetics. 2017 May 8;9:49. doi: 10.1186/s13148-017-0349-z (PMC5422977; doi:10.1186/s13148-017-0349-z)
Supplement: Additional file 1: Figure S1. — Flowchart of the study participants and sample attrition. (PPTX 38 kb) [file 13148_2017_349_MOESM1_ESM.pptx]

## Slide 1
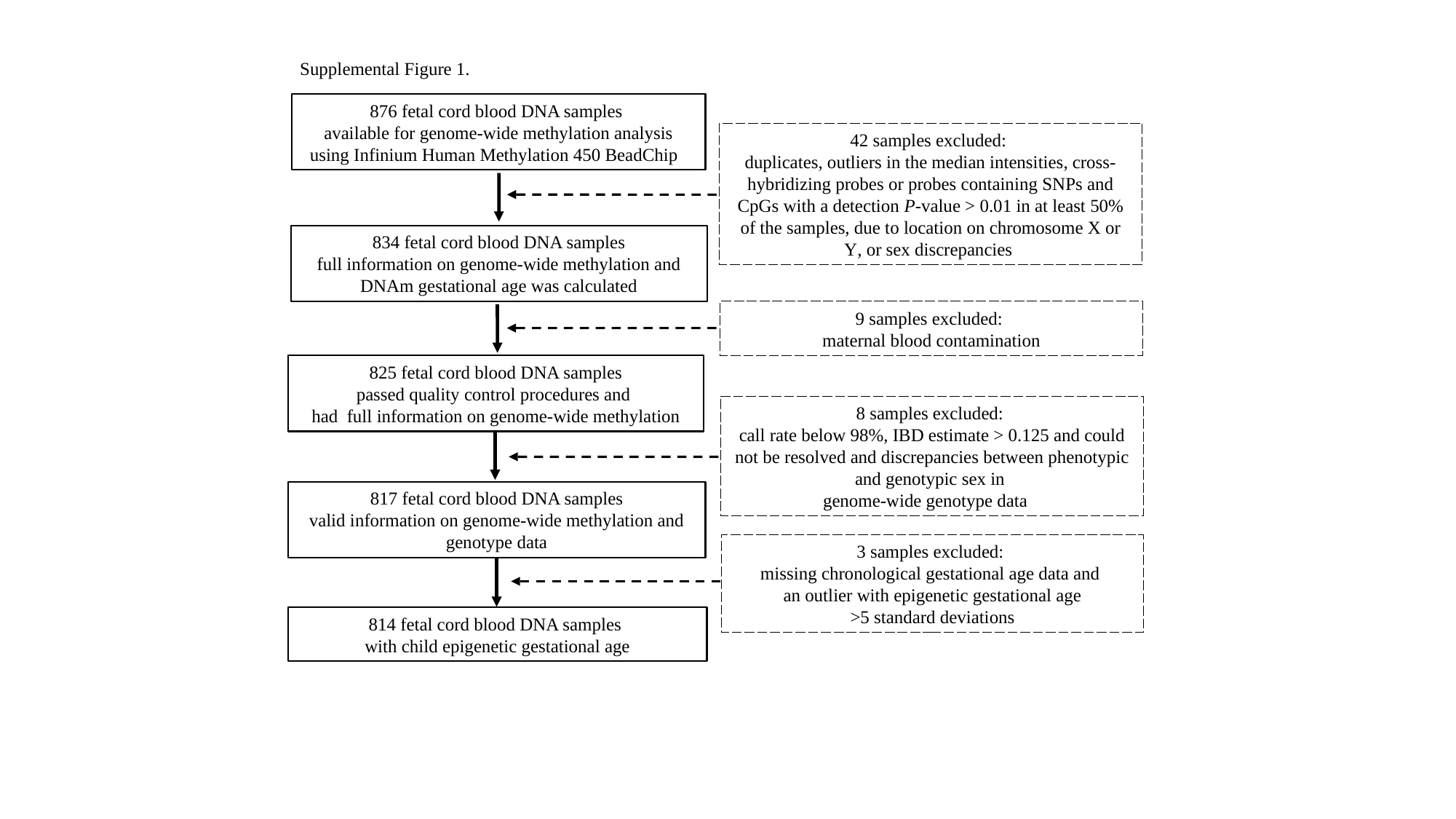

Supplemental Figure 1.
876 fetal cord blood DNA samples
available for genome-wide methylation analysis using Infinium Human Methylation 450 BeadChip
42 samples excluded:
duplicates, outliers in the median intensities, cross-hybridizing probes or probes containing SNPs and CpGs with a detection P-value > 0.01 in at least 50% of the samples, due to location on chromosome X or Y, or sex discrepancies
834 fetal cord blood DNA samples
full information on genome-wide methylation and DNAm gestational age was calculated
9 samples excluded:
maternal blood contamination
825 fetal cord blood DNA samples
passed quality control procedures and
had full information on genome-wide methylation
8 samples excluded:
call rate below 98%, IBD estimate > 0.125 and could not be resolved and discrepancies between phenotypic and genotypic sex in
genome-wide genotype data
817 fetal cord blood DNA samples
valid information on genome-wide methylation and genotype data
3 samples excluded:
missing chronological gestational age data and
an outlier with epigenetic gestational age
 >5 standard deviations
814 fetal cord blood DNA samples
with child epigenetic gestational age
