# Supplementary material for: Associations between maternal risk factors of adverse pregnancy and birth outcomes and the offspring epigenetic clock of gestational age at birth
Source: Clin Epigenetics. 2017 May 8;9:49. doi: 10.1186/s13148-017-0349-z (PMC5422977; doi:10.1186/s13148-017-0349-z)
Supplement: Additional file 2: Table S1. — Associations between maternal characteristics during pregnancy and offspring DNAm GA at birth based on cord blood methylation data. Table S2. Associations between maternal characteristics during pregnancy and offspring DNAm GA at birth based on cord blood methylation data when additionally adjusting for offspring birth weight SD score at birth*. Table S3. Associations between maternal characteristics and the offspring Horvath epigenetic age at birth based on cord blood methylation data*. Table S4. Associations between offspring characteristics and DNAm GA at birth based on cord blood methylation data. Table S5. Associations between offspring characteristics and the offspring Horvath epigenetic age at birth based on cord blood methylation data*. (DOCX 65 kb) [file 13148_2017_349_MOESM2_ESM.docx]

Additional file 2

| **Table S1. Associations between maternal characteristics during pregnancy and offspring DNAm GA at birth based on cord blood methylation data.** | | | | | | |
| --- | --- | --- | --- | --- | --- | --- |
| **Maternal characteristics** | **DNAm GA difference*** | | | **DNAm GA residual*** | | |
|  | DNAm GAA /GAD in weeks | 95% Confidence Interval | p-value  ** | DNAm GAA /GAD in SD units | 95% Confidence Interval | p-value  ** |
| **Pre-pregnancy risk factors** |  |  |  |  |  |  |
| Maternal age at delivery, years | 0.02 | -0.004, 0.04 | 0.11 | 0.01 | -0.02, 0.02 | 0.10 |
| Below 20 years (0=no,  1=yes) | -0.02 | -0.78, 0.74 | 0.96 | 0.18 | -0.25, 0.60 | 0.41 |
| Above 40 years (0=no,  1=yes) | **0.44** | **0.09, 0.80** | **0.01** | **0.30** | **0.10, 0.50** | **0.003** |
| Pre-eclampsia in a previous pregnancy (0=no, 1=yes) | **0.31** | **0.003, 0.61** | **0.05** | 0.07 | -0.10, 0.23 | 0.44 |
| Intrauterine growth restriction in previous pregnancy (0=no, 1=yes) | -0.01 | -0.42, 0.40 | 0.97 | -0.08 | -0.30, 0.15 | 0.51 |
| Gestational diabetes in previous pregnancy (0=no, 1=yes) | -0.21 | -0.62, 0.20 | 0.32 | -0.16 | -0.39, 0.06 | 0.16 |
| Diet treated (0=no, 1=yes) | -0.05 | -0.48, 0.38 | 0.83 | -0.07 | -0.31, 0.17 | 0.56 |
| Insulin treated (0=no, 1=yes) | **-1.44** | **-2.63, -0.24** | **0.02** | **-0.84** | **-1.50, -0.19** | **0.01** |
| Pre-pregnancy body mass index, kg/m^2^ | -0.02 | -0.04, 0.004 | 0.11 | -0.007 | -0.02, 0.004 | 0.19 |
| ≥ 30 kg/m^2^ (0=no, 1=yes) | -0.15 | -0.42, 0.11 | 0.26 | -0.03 | -0.17, 0.12 | 0.70 |
| Pre-pregnancy chronic hypertension (0=no, 1=yes) | 0.003 | -0.36, 0.37 | 0.99 | -0.09 | -0.29, 0.11 | 0.38 |
| Pre-pregnancy type 1 diabetes (0=no, 1=yes) | 0.49 | -0.56, 1.52 | 0.36 | -0.29 | -0.86, 0.28 | 0.32 |
| Pre-pregnancy systemic lupus erythematosus (0=no, 1=yes) | 0.97 | -1.53, 3.48 | 0.45 | 0.22 | -1.17, 1.61 | 0.75 |
| Sjögren’s syndrome (0=no, 1=yes) | -0.91 | -1.98, 0.17 | 0.10 | **-0.63** | **-1.22, -0.03** | **0.04** |
| Previous pregnancy with fetal demise (>22 gestational weeks or over 500 g) (0=no, 1=yes) | **0.82** | **0.13, 1.50** | **0.02** | 0.37 | -0.006, 0.75 | 0.054 |
| Number of known pre-pregnancy risk factors |  |  |  |  |  |  |
| No known pre-pregnancy  risk factors | Ref |  |  | Ref |  |  |
| 1 or 2 pre-pregnancy  risk factors | 0.23 | -0.19, 0.66 | 0.28 | 0.08 | -0.15, 0.32 | 0.48 |
| 3 or more pre-  pregnancy risk factors | **1.08** | **0.33, 1.83** | **0.005** | 0.37 | -0.05, 0.78 | 0.08 |
| **Pregnancy disorders** |  |  |  |  |  |  |
| Gestational diabetes (0=no, 1=yes) | 0.06 | -0.24, 0.36 | 0.68 | 0.01 | -0.15, 0.18 | 0.87 |
| Gestational diabetes treatment |  |  |  |  |  |  |
| No gestational diabetes | Ref |  |  | Ref |  |  |
| Diet treated | 0.12 | -0.20, 0.45 | 0.46 | 0.04 | -0.14, 0.22 | 0.66 |
| Insulin treated | -0.12 | -0.73, 0.49 | 0.70 | -0.18 | -0.51, 0.16 | 0.30 |
| Hypertension spectrum pregnancy disorders |  |  |  |  |  |  |
| No hypertension spectrum  pregnancy disorder | Ref |  |  | Ref |  |  |
| Gestational hypertension | 0.02 | -0.40, 0.44 | 0.93 | 0.002 | -0.23, 0.24 | 0.98 |
| Pre-eclampsia | **0.75** | **0.27, 1.22** | **0.002** | 0.06 | -0.20, 0.33 | 0.64 |
| Early | **1.91** | **0.63, 3.20** | **0.004** | -0.04 | -0.74, 0.65 | 0.90 |
| Late | **0.58** | **0.07, 1.09** | **0.02** | 0.08 | -0.20, 0.36 | 0.58 |
| Non-severe | 0.19 | -0.37, 0.75 | 0.50 | -0.08 | -0.39, 0.24 | 0.63 |
| Severe | **1.99** | **1.17, 2.81** | **2*10^-6^** | 0.37 | -0.09, 0.83 | 0.11 |
| Chronic hypertension*** | -0.03 | -0.37, 0.32 | 0.88 | -0.06 | -0.25, 0.13 | 0.54 |
| **Other characteristics** |  |  |  |  |  |  |
| Education level |  |  |  |  |  |  |
| Lower secondary or less | Ref |  |  | Ref |  |  |
| Upper secondary | 0.05 | -0.13, 0.22 | 0.61 | 0.07 | -0.25, 0.39 | 0.67 |
| Tertiary | -0.01 | -0.17, 0.15 | 0.88 | -0.03 | -0.32, 0.27 | 0.86 |
| Parity (0=primiparous, 1=multiparous) | 0.10 | -0.17, 0.37 | 0.47 | -0.02 | -0.17, 0.13 | 0.84 |
| Smoking during pregnancy |  |  |  |  |  |  |
| Non-smoker | Ref |  |  | Ref |  |  |
| Quit during first trimester | 0.15 | -0.56, 0.84 | 0.70 | 0.04 | -0.35, 0.44 | 0.82 |
| Smoked throughout the  pregnancy | 0.10 | -1.15, 1.36 | 0.87 | -0.04 | -0.73, 0.66 | 0.91 |
| Alcohol use during pregnancy (0=no, 1=yes) | -0.06 | -0.43, 0.30 | 0.73 | -0.009 | -0.21, 0.19 | 0.93 |
| Mode of delivery (0=vaginal, 1=caesarean) | 0.04 | -0.28, 0.36 | 0.83 | -0.01 | -0.18, 0.16 | 0.94 |
| Antenatal betamethasone treatment (0=no, 1=yes) | **1.25** | **0.62, 1.87** | **0.0001** | 0.06 | -0.28, 0.40 | 0.72 |
| Timing of antenatal betamethasone treatment |  |  |  |  |  |  |
| No betamethasone  treatment | Ref |  |  | Ref |  |  |
| 30 days or less before  delivery | **2.73** | **1.75, 3.71** | **6*10^-8^** | 0.21 | -0.32, 0.74 | 0.47 |
| 30 days or more before  delivery | 0.96 | -0.02, 1.93 | 0.054 | 0.21 | -0.32, 0.74 | 0.44 |
| *All analyses were adjusted for cell-type composition and population stratification estimated with 2 multi-dimensional scaling components based on genome-wide data.  **p-value <0.0029 indicates a Bonferroni-corrected p-value < 0.05 assuming tests with 17 main variables.  ***This category includes 109 women with pre-pregnancy chronic hypertension and 25 women with hypertension detected before the 20^th^ gestational week in the index pregnancy. | | | | | | |

| **Table S2.** **Associations between maternal characteristics during pregnancy and offspring DNAm GA at birth based on cord blood methylation data when additionally adjusting for offspring birth weight SD score at birth.*** | | | | | | |
| --- | --- | --- | --- | --- | --- | --- |
| **Maternal characteristics** | **DNAm GA difference*** | | | **DNAm GA residual*** | | |
|  |  | | |  | | |
|  | DNAm GAA /GAD in weeks | 95% Confidence Interval | p-value  ** | DNAm GAA /GAD in SD units | 95% Confidence Interval | p-value  ** |
| **Pre-pregnancy risk factors** |  |  |  |  |  |  |
| Maternal age at delivery, years | 0.02 | -0.006, 0.04 | 0.15 | 0.01 | -0.002, 0.02 | 0.11 |
| Below 20 years (0=no,  1=yes) | 0.05 | -0.70, 0.79 | 0.90 | 0.17 | -0.25, 0.60 | 0.42 |
| Above 40 years (0=no,  1=yes) | **0.51** | **0.16, 0.86** | **0.004** | **0.30** | **0.10, 0.50** | **0.003** |
| Pre-eclampsia in previous pregnancy (0=no, 1=yes) | 0.27 | -0.02,0.56 | 0.08 | 0.06 | -0.10, 0.23 | 0.46 |
| Intrauterine growth restriction in previous pregnancy (0=no, 1=yes) | -0.29 | -0.70, 0.12 | 0.16 | -0.08 | -0.31, 0.15 | 0.51 |
| Gestational diabetes in previous pregnancy (0=no, 1=yes) | -0.07 | -0.48, 0.33 | 0.72 | -0.16 | -0.39, 0.06 | 0.16 |
| Diet treated (0=no, 1=yes) | 0.08 | -0.35, 0.50 | 0.72 | -0.07 | -0.31, 0.17 | 0.58 |
| Insulin treated (0=no, 1=yes) | **-1.23** | **-2.40, -0.05** | **0.04** | **-0.87** | **-1.53, -0.21** | **0.01** |
| Pre-pregnancy body mass index, kg/m^2^ | -0.005 | -0.02, 0.01 | 0.61 | -0.007 | -0.02, 0.003 | 0.17 |
| ≥ 30 kg/m^2^ (0=no, 1=yes) | 0.01 | -0.25, 0.28 | 0.92 | -0.03 | -0.18, 0.12 | 0.72 |
| Pre-pregnancy chronic hypertension (0=no, 1=yes) | -0.17 | -0.54, 0.19 | 0.35 | -0.09 | -0.29, 0.11 | 0.39 |
| Pre-pregnancy type 1 diabetes (0=no, 1=yes) | 0.50 | -0.52, 1.52 | 0.34 | -0.29 | -0.86, 0.28 | 0.31 |
| Pre-pregnancy systemic lupus erythematosus (0=no, 1=yes) | 0.84 | -1.61, 3.29 | 0.50 | 0.22 | -1.17, 1.61 | 0.75 |
| Sjögren’s syndrome (0=no, 1=yes) | **-1.14** | **-2.19, -0.08** | **0.03** | **-0.62** | **-1.22, -0.02** | **0.04** |
| Previous pregnancy with fetal demise (>22 gestational weeks or over 500 g) (0=no, 1=yes) | **0.95** | **0.28, 1.62** | **0.006** | 0.36 | -0.01, 0.74 | 0.06 |
| Number of known pre-pregnancy risk factors |  |  |  |  |  |  |
| No known pre-pregnancy  risk factors | Ref |  |  | Ref |  |  |
| 1 or 2 pre-pregnancy  risk factors | 0.36 | -0.05, 0.78 | 0.09 | 0.08 | -0.15, 0.32 | 0.48 |
| 3 or more pre-  pregnancy risk factors | **1.18** | **0.45, 1.92** | **0.001** | 0.37 | -0.05, 0.79 | 0.08 |
| **Pregnancy disorders** |  |  |  |  |  |  |
| Gestational diabetes (0=no, 1=yes) | -0.004 | -0.04, 0.03 | 0.82 | 0.01 | -0.15, 0.18 | 0.89 |
| Gestational diabetes treatment |  |  |  |  |  |  |
| No gestational diabetes | Ref |  |  | Ref |  |  |
| Diet treated | 0.24 | -0.08, 0.56 | 0.14 | 0.05 | -0.13, 0.23 | 0.58 |
| Insulin treated | -0.01 | -0.61, 0.58 | 0.96 | -0.17 | -0.51, 0.17 | 0.32 |
| Hypertension spectrum pregnancy disorders |  |  |  |  |  |  |
| No hypertension spectrum  pregnancy disorder | Ref |  |  | Ref |  |  |
| Gestational hypertension | 0.03 | -0.38, 0.45 | 0.87 | 0.001 | -0.23, 0.24 | 0.99 |
| Pre-eclampsia | **0.50** | **0.02, 0.98** | **0.04** | 0.07 | -0.20, 0.34 | 0.62 |
| Early | 1.04 | -0.26, 2.34 | 0.11 | -0.01 | -0.74, 0.71 | 0.97 |
| Late | **0.51** | **0.01, 1.01** | **0.05** | 0.12 | -0.16, 0.41 | 0.40 |
| Non-severe | 0.20 | -0.36, 0.76 | 0.48 | -0.04 | -0.36, 0.28 | 0.79 |
| Severe | **1.37** | **0.53, 2.22** | **0.002** | 0.42 | -0.06, 0.90 | 0.09 |
| Chronic hypertension*** | -0.11 | -0.45, 0.22 | 0.51 | -0.05 | -0.24, 0.14 | 0.58 |
| **Other characteristics** |  |  |  |  |  |  |
| Education level |  |  |  |  |  |  |
| Lower secondary or less | Ref |  |  | Ref |  |  |
| Upper secondary | 0.04 | -0.27, 0.35 | 0.63 | 0.04 | -0.13, 0.22 | 0.63 |
| Tertiary | -0.07 | -0.36, 0.22 | 0.62 | -0.01 | -0.18, 0.15 | 0.87 |
| Parity (0=primiparous, 1=multiparous) | 0.11 | 0.16, 0.38 | 0.41 | -0.02 | -0.17, 0.13 | 0.84 |
| Smoking during pregnancy |  |  |  |  |  |  |
| Non-smoker | Ref |  |  | Ref |  |  |
| Quit during first trimester | 0.15 | -0.55, 0.85 | 0.67 | 0.09 | -0.31, 0.49 | 0.65 |
| Smoked throughout the  pregnancy | -0.15 | -1.38, 1.09 | 0.82 | -0.04 | -0.74, 0.66 | 0.92 |
| Alcohol use during pregnancy (0=no, 1=yes) | -0.04 | -0.40 0.32 | 0.83 | -0.007 | -0.21, 0.20 | 0.94 |
| Mode of delivery (0=vaginal, 1=caesarean) | 0.08 | -0.23, 0.39 | 0.62 | -0.01 | -0.17, 0.16 | 0.94 |
| Antenatal betamethasone treatment (0=no, 1=yes) | **0.80** | **0.16, 1.45** | **0.01** | 0.08 | -0.28, 0.43 | 0.67 |
| Timing of antenatal betamethasone treatment |  |  |  |  |  |  |
| No betamethasone  treatment | Ref |  |  | Ref |  |  |
| 30 days or less before  delivery | **1.97** | **0.94, 2.99** | **0.0002** | 0.29 | -0.29, 0.84 | 0.34 |
| 30 days or more before  delivery | 0.81 | -0.16, 1.77 | 0.10 | 0.28 | -0.33, 0.77 | 0.43 |
| *All analyses were adjusted for cell-type composition and population stratification estimated with 2 multi-dimensional scaling components based on genome-wide data and birth weight SD score according to Finnish growth references (23).  **p-value <0.0029 indicates a Bonferroni-corrected p-value < 0.05 assuming tests with 17 main variables.  ***This category includes 109 women with pre-pregnancy chronic hypertension and 25 women with hypertension detected before 20^th^ gestational week in the index pregnancy. | | | | | | |

| **Table S3. Associations between maternal characteristics and the offspring Horvath epigenetic age at birth based on cord blood methylation data.*** | | | |
| --- | --- | --- | --- |
| **Maternal characteristics** | Epigenetic age acceleration/deceleration in weeks | 95% Confidence Interval | p-value** |
| **Pre-pregnancy risk factors** |  |  |  |
| Maternal age at delivery, years | -0.13 | -0.35, 0.10 | 0.26 |
| Below 20 years (0=no,  1=yes) | 3.26 | -4.71, 11.22 | 0.42 |
| Above 40 years (0=no,  1=yes) | 2.09 | -1.65, 5.82 | 0.27 |
| Pre-eclampsia in previous pregnancy (0=no, 1=yes) | -0.40 | -3.57, 2.78 | 0.81 |
| Intrauterine growth restriction in previous pregnancy (0=no, 1=yes) | 2.22 | -2.07, 6.51 | 0.31 |
| Gestational diabetes in previous pregnancy (0=no, 1=yes) | 2.22 | -2.06, 6.50 | 0.31 |
| Diet treated (0=no, 1=yes) | 1.17 | -3.33, 5.66 | 0.61 |
| Insulin treated (0=no, 1=yes) | 9.96 | -2.55, 22,47 | 0.12 |
| Pre-pregnancy body mass index, kg/m^2^ | -0.08 | -0.13, 0.29 | 0.44 |
| ≥ 30 kg/m^2^ (0=no, 1=yes) | -0.86 | -3.62, 1.91 | 0.54 |
| Pre-pregnancy chronic hypertension (0=no, 1=yes) | -1.54 | -5.37, 2.30 | 0.43 |
| Pre-pregnancy type 1 diabetes (0=no, 1=yes) | **-13.10** | **-23.97, -2.22** | **0.02** |
| Pre-pregnancy systemic lupus erythematosus (0=no, 1=yes) | -9.48 | -35.64, 16.68 | 0.47 |
| Pre-pregnancy Sjögren’s syndrome (0=no, 1=yes) | -5.01 | -16.24, 6.22 | 0.38 |
| Previous pregnancy with fetal demise (>22 gestational weeks or over 500 g) (0=no, 1=yes) | 3.55 | -3.61, 10.72 | 0.33 |
| Number of known pre-pregnancy risk factors |  |  |  |
| No known pre-pregnancy  risk factors | Ref |  |  |
| 1 or 2 pre-pregnancy risk factors | -1.72 | -6.16, 2.72 | 0.45 |
| 3 or more pre-pregnancy  risk factors | -2.38 | -10.23, 5.48 | 0.55 |
| **Pregnancy disorders** |  |  |  |
| Gestational diabetes (0=no, 1=yes) | 1.51 | -1.62, 4.63 | 0.34 |
| Gestational diabetes treatment |  |  |  |
| No gestational diabetes | Ref |  |  |
| Diet treated | 1.28 | -2.15, 4.70 | 0.46 |
| Insulin treated | 1.95 | -4.39, 8.30 | 0.60 |
| Hypertension spectrum pregnancy disorders |  |  |  |
| No hypertension spectrum  pregnancy disorder | Ref |  |  |
| Gestational hypertension | -3.55 | -7.96, 0.87 | 0.12 |
| Pre-eclampsia | 2.83 | -2.19, 7.85 | 0.27 |
| Early | 18.20 | 4.79, 31.62 | 0.008 |
| Late | 1.55 | -3.79, 6.88 | 0.57 |
| Non-severe | 1.36 | -4.60, 7.33 | 0.65 |
| Severe | 8.69 | 0.05, 17.34 | 0.05 |
| Chronic hypertension*** | -2.29 | -5.86, 1.29 | 0.21 |
| **Other characteristics** |  |  |  |
| Education level |  |  |  |
| Lower secondary or less | Ref |  |  |
| Upper secondary | 0.77 | -2.62, 4.16 | 0.66 |
| Tertiary | -0.52 | -3.65, 2.60 | 0.74 |
| Parity (0=primiparous, 1=multiparous) | **-2.93** | **-5.80, -0.05** | **0.05** |
| Smoking during pregnancy |  |  |  |
| Non-smoker | Ref |  |  |
| Quit during first trimester | 2.10 | -5.30, 9.50 | 0.58 |
| Smoked throughout the pregnancy | -1.27 | -14.44, 11.91 | 0.85 |
| Alcohol use during pregnancy (0=no, 1=yes) | 1.61 | -2.12, 5.34 | 0.40 |
| Mode of delivery (0=vaginal, 1=caesarean) | 1.23 | -2.08, 4.55 | 0.47 |
| Antenatal betamethasone treatment (0=no, 1=yes) | -0.64 | -7.20, 5.92 | 0.85 |
| Timing of antenatal betamethasone treatment |  |  |  |
| No betamethasone  treatment | Ref |  |  |
| 30 days or less before birth  delivery | -1.08 | -11.55, 9.40 | 0.84 |
| 30 days or more before  delivery | 2.97 | -6.46, 14.39 | 0.46 |
| *All analyses were adjusted for cell-type composition and population stratification estimated with 2 multi-dimensional scaling components based on genome-wide data.  **p-value <0.0029 indicates a Bonferroni-corrected p-value < 0.05 assuming tests with 17 main variables.  ***This category includes 111 women with pre-pregnancy chronic hypertension and 24 women with hypertension detected before 20^th^ gestational week in the index pregnancy. | | | |

| **Table S4. Associations between offspring characteristics and DNAm GA at birth based on cord blood methylation data.** | | | | | | |
| --- | --- | --- | --- | --- | --- | --- |
|  | **DNAm GA difference*** | | | **DNAm GA residual*** | | |
| **Offspring characteristics** | DNAm GAA /GAD in weeks | 95% Confidence Interval | p-value  ** | DNAm GAA /GAD in SD units | 95% Confidence Interval | p-value  ** |
| Child sex (0=boys, 1=girls) | **0.30** | **0.05, 0.55** | **0.02** | **0.16** | **0.02, 0.30** | **0.02** |
| Birth weight, kg | **-0.67** | **-0.90, -0.44** | **3*10^-9^** | 0.03 | -0.10, 0.16 | 0.66 |
| Small for gestational age (0=no, 1=yes)*** | **1.08** | **0.33, 1.83** | **0.005** | -0.04 | -0.45, 0.37 | 0.84 |
| Birth length, cm | **-0.15** | **-0.21, -0.10** | **9*10^-9^** | 0.01 | -0.02. 0.04 | 0.48 |
| Small for gestational age  (0=no, 1=yes)*** | 0.22 | -0.56, 1.0 | 0.58 | -0.21 | -0.64, 0.23 | 0.35 |
| Head circumference, cm | **-0.18** | **-0.26, -0.10** | **2*10^-6^** | 0.01 | -0.03, 0.06 | 0.57 |
| Small for gestational age  (0=no, 1=yes)*** | -0.13 | -1.08., 0.82 | 0.79 | -0.11 | -0.64, 0.41 | 0.68 |
| Ponderal index, kg/m^3^ | **-0.05** | **-0.10, -0.01** | **0.01** | -0.006 | -0.03, 0.02 | 0.66 |
| Placenta weight, g | **-0.15** | **-0.25, -0.06** | **0.002** | -0.0004 | -0.05, 0.05 | 0.99 |
| Cord blood pH |  |  |  |  |  |  |
| Arterial | 0.82 | -0.92, 2.56 | 0.35 | 0.09 | -0.75, 0.94 | 0.83 |
| Venous | 2.05 | -1.73, 5.82 | 0.28 | 0.08 | -1.60, 1.77 | 0.92 |
| Apgar score |  |  |  |  |  |  |
| 9-10 | Ref |  |  | Ref |  |  |
| 7-8 | 0.10 | -0.23, 0.43 | 0.57 | -0.01 | -0.19, 0.17 | 0.89 |
| ≤ 6 | **0.72** | **0.19, 1.27** | **0.009** | **0.37** | **0.07, 0.67** | **0.01** |
| *All analyses were adjusted for cell-type composition and population stratification estimated with 2 multi-dimensional scaling components based on genome-wide data; Anthropometric data were adjusted for sex.  ** p-value <0.0055 indicates a Bonferroni-corrected p-value < 0.05 assuming tests with 9 main variables.  ***Small for gestational age indicates birth size for sex and gestational age SD ≤ -2 according to Finnish growth references (23). | | | | | | |

| **Table S5. Associations between offspring characteristics and the offspring Horvath epigenetic age at birth based on cord blood methylation data.*** | | | |
| --- | --- | --- | --- |
| **Offspring characteristics** | Epigenetic age acceleration/deceleration in weeks | 95% Confidence Interval | p-value** |
| Child sex (0=boys, 1=girls) | 2.46 | -0.18, 5.10 | 0.07 |
| Birth weight, kg | -0.74 | -3.20, 1.71 | 0.55 |
| Small for gestational age (0=no, 1=yes)*** | 2.57 | -5.32, 10.45 | 0.52 |
| Birth length, cm | 0.06 | -0.52, 0.65 | 0.84 |
| Small for gestational age  (0=no, 1=yes)*** | 5.03 | -3.16, 13.22 | 0.23 |
| Head circumference, cm | -0.44 | -0.28, -0.40 | 0.30 |
| Small for gestational age  (0=no, 1=yes)*** | 4.44 | -5.58, 14.46 | 0.38 |
| Ponderal index, kg/m^3^ | -0.37 | -0.85, -0.11 | 0.13 |
| Placenta weight, g | -0.81 | -1.79, 0.16 | 0.10 |
| Cord blood pH |  |  |  |
| Arterial | 1.76 | -16.07, 19.59 | 0.84 |
| Venous | **67.93** | **19.69, 119.17** | **0.006** |
| Apgar score |  |  |  |
| 9-10 | Ref |  |  |
| 7-8 | -1.34 | -4.84, 2.17 | 0.45 |
| ≤ 6 | -3.54 | -9.20, 2.13 | 0.22 |
| *All analyses were adjusted for cell-type composition and population stratification estimated with 2 multi-dimensional scaling components based on genome-wide data; Anthropometric data were adjusted also for child sex.  ** p-value <0.0055 indicates a Bonferroni-corrected p-value < 0.05 assuming tests with 9 main variables.  ***Small for gestational age indicates birth size for sex and gestational age SD ≤ -2 according to Finnish growth references (23). | | | |
